# Gleanings in Science and Medicine

**Published:** 1893-04-22

**Authors:** 


### GLEANINGS IN SCIENCE AND MEDICINE.

Proof readers, to be experts of the highest value, need not be linguists. The American Bible Society has acquired the services of a proof-reader in 242 languages. What he reads he reads by comparison, for Mr. Emery McLoan understands but one language, and that is English. The task is one involving great tension of eye and thought. The wages of such readers are regulated by "the union," though there is no financial increase in the salary according to the number of languages thus read in.

The key to the history of a nation is in its grammar, in its spelling. The changes through which a country passes, leave the stamp of an outside influence upon her speech. Take our own country as an instance of this. A glance will prove how deeply marked upon our language are the great periods of the incursion of outside nations. The struggle for supremacy of speech was included in the struggle between race and race. Conqueror and conquered continued their battles in our words. Instances without number arise on all hands to prove this, and wherein even the compromises arrived at between the two are discernable. In the names of the days of our weeks, for example, the Saxon element is paramount, whilst the Norman influence dominates the months of the year. Again, such animals as we eat, have Saxon names, until they have passed through the slaughter-house, when they become Norman—thus oxen alive is beef when dead, sheep is mutton, calf is veal, and so on. All the old historic landmarks of our language will be lost sight of if the wish for a radical change in our language gains ground as, unluckily, it is fast doing now. Euphonistic spelling is but a sign of the times, the love of altering all things. It is quite true that it would obviate many of our existing difficulties, if all words were spelt "as they sounded," but for such trivial ends it is barely worth while to sacrifice the marks of the past, which should be sacred to us, as being of great historic evidence and value.

The visitors to the World's Great Fair are to have their creature comforts conscientiously attended to, for not only will there be a perfectly equipped hospital, demonstrating the latest theories of the day, but added to this central establishment there will be also numerous movable hospitals, or relief stations, which are placed at given distances in the grounds. It is only likely that the heat, the crushing, and fatigue will find many only too ready to benefit by this forethought on the part of the Medical Directorate of the Exhibition.

The language of medicine, in its severely scientific form, is a sealed book to the uninitiated. This is significantly illustrated in the pages of *Le Progrès Médical*, which journal has lately published a list of some comparatively new drugs and their scientific names, of which we append a specimen:—

| Common Names. | Scientific Names. |
|---|---|
| Antipyrine, Analgesine | Phenyldunelhylpqrazoline. |
| Antinervine | Salicylbromanilide. |
| Antisepsene | Paramonobromophenylaectamide. |
| Bromol | Tribromphenol. |
| Orexine | Phenyldehydroquinazolinehyrdrochloride. |
| Primuline | Sodium-thioparatoluidinesulphonate. |
| Sulphonal | Dielhylsulphondmethylethane. |

